# Supplementary material for: From Crisis To Crisis: Impacts Of The COVID-19 Pandemic On People Living With HIV And AIDS Service Organizations In Indiana
Source: Res Sq. 2022 Feb 21:rs.3.rs-1003567. Preprint. [Version 1] doi: 10.21203/rs.3.rs-1003567/v1 (PMC8863148; doi:10.21203/rs.3.rs-1003567/v1)
Supplement: Supplement 1 [file 5211be11e1bdd72f641bc558.pdf]

HIV Questionnaire:

Q1: What does your organization do as a whole? What is your role specifically within the organization?

Q2: Under normal circumstances (pre-COVID), what specific services/resources does your organization provide for individuals with HIV?

Potential Probes

- What does first contact/outreach typically look-like?
- What is the typical workflow of the intake process?
- How is an individual referred to other internal programs or external programs?
- How long does an individual usually use your services? Why would an individual usually fall out of service, and if so, what does your organization do to bring this person back into service?
- What kinds of testing do you normally offer, and how and where does it normally occur?

Q3: Did COVID change things for your organization? If yes, how?

Potential Probes:

- How did your daily interactions and communication with clients change?
- How did the day-to-day lives of clients change as a result of the pandemic?
    - Did you notice any specific changes in the needs/behavior of clients?
- Did your organization have the proper resources (structural, human, physical, financial) to address any new needs?
- Were there any difficulties in communicating crucial information/guidelines to clients?
    - If so, are there any resources that would help alleviate such difficulties?

Q4: How prepared was your organization to deal with COVID?

Potential Probes:

- How efficient and effective were [local, state, federal] governmental responses?
    - What worked well? What could be improved?
- How prepared were your local or statewide partners?
    - What worked well? What could be improved?
- Could any changes implemented during COVID be carried over into the future?
- Are you concerned about a potential influx of people in need of your organization's resources after eviction freezes are lifted?
    - How is your organization preparing?

Q5: Did COVID-related policy changes (Indiana phases) affect your organization? If yes, how did it affect your organization's ability to function?

Potential Probes:

- Was your organization able to obtain additional funding as a result of new policies?
- How many significant changes came from the [local, state, federal level]?
- Were there any policies that were unreasonable or unattainable; if so, what would have changed such an outcome?
- Are there any standards that were not, but should have been implemented as official policy?

Q6: Can we contact you in the future if we have additional questions?
